# Costs and expected gain in lifetime health from intensive care versus general ward care of 30,712 individual patients: a distribution-weighted cost-effectiveness analysis

**DOI:** 10.1186/s13054-017-1792-0

**Published:** 2017-08-21

**Authors:** Frode Lindemark, Øystein A. Haaland, Reidar Kvåle, Hans Flaatten, Ole F. Norheim, Kjell A. Johansson

**Affiliations:** 1 Department of Research and Development, Haukeland University Hospital, Bergen, Norway; 2 Department of Global Public Health and Primary Care, University of Bergen, Bergen, Norway; 3 Norwegian Intensive Care Registry, Helse Bergen HF, Bergen, Norway; 4 Department of Anesthesia and Intensive Care, Haukeland University Hospital, Bergen, Norway; 5 Department of Clinical Medicine, University of Bergen, Bergen, Norway

**Keywords:** Intensive care, Cost-effectiveness, Severity of disease, Quality-adjusted life years, Resource allocation, Health priorities

## Abstract

**Background:**

Clinicians, hospital managers, policy makers, and researchers are concerned about high costs, increased demand, and variation in priorities in the intensive care unit (ICU). The objectives of this modelling study are to describe the extra costs and expected health gains associated with admission to the ICU versus the general ward for 30,712 patients and the variation in cost-effectiveness estimates among subgroups and individuals, and to perform a distribution-weighted economic evaluation incorporating extra weighting to patients with high severity of disease.

**Methods:**

We used a decision-analytic model that estimates the incremental cost per quality-adjusted life year (QALY) gained (ICER) from ICU admission compared with general ward care using Norwegian registry data from 2008 to 2010. We assigned increasing weights to health gains for those with higher severity of disease, defined as less expected lifetime health if not admitted. The study has inherent uncertainty of findings because a randomized clinical trial comparing patients admitted or rejected to the ICU has never been performed. Uncertainty is explored in probabilistic sensitivity analysis.

**Results:**

The mean cost-effectiveness of ICU admission versus ward care was €11,600/QALY, with 1.6 QALYs gained and an incremental cost of €18,700 per patient. The probability (*p*) of cost-effectiveness was 95% at a threshold of €22,000/QALY. The mean ICER for medical admissions was €10,700/QALY (*p* = 97%), €12,300/QALY (*p* = 93%) for admissions after acute surgery, and €14,700/QALY (*p* = 84%) after planned surgery. For individualized ICERs, there was a 50% probability that ICU admission was cost-effective for 85% of the patients at a threshold of €64,000/QALY, leaving 15% of the admissions not cost-effective. In the distributional evaluation, 8% of all patients had distribution-weighted ICERs (higher weights to gains for more severe conditions) above €64,000/QALY. High-severity admissions gained the most, and were more cost-effective.

**Conclusions:**

On average, ICU admission versus general ward care was cost-effective at a threshold of €22,000/QALY (*p* = 95%). According to the individualized cost-effectiveness information, one in six ICU admissions was not cost-effective at a threshold of €64,000/QALY. Almost half of these admissions that were not cost-effective can be regarded as acceptable when weighted by severity of disease in terms of expected lifetime health. Overall, existing ICU services represent reasonable resource use, but considerable uncertainty becomes evident when disaggregating into individualized results.

**Electronic supplementary material:**

The online version of this article (doi:10.1186/s13054-017-1792-0) contains supplementary material, which is available to authorized users.

## Background

Clinicians, hospital managers, policy makers, and researchers are concerned about high costs, increased demand, and variation in priorities in intensive care [1–7]. Population-level studies find that, on average, admission of critically ill patients to intensive care units (ICUs) versus non-intensive care is cost-effective [8–10]. It does not necessarily follow that every individual ICU admission represents efficient resource use; the critically ill are a diverse group of patients [11]. However, certain characteristics among the patients may justify higher resource use. Society may accept higher costs per unit of health gain among severe cases compared to less severe cases [12–14]. A better understanding of individual variation in terms of severity of disease, cost, and effects of ICUs is therefore needed.

Analysis of the extra costs and health gains associated with ICU admission versus some next-best treatment option for the critically ill could inform ICU priorities [15, 16]. Robust estimates of the real health benefits and extra costs of ICU admission are unavailable because the alternative to ICU admission—the appropriate comparator—is hard to quantify since randomised controlled trials (RCTs) cannot be performed due to ethical reasons [9, 15, 17]. Previous cost-effectiveness analyses (CEAs) of intensive care have used average cost-effectiveness ratios or assumed that patients incur no costs and die if not admitted to the ICU [8, 18–20]. Two cost-effectiveness analyses from Europe compared ICU admission to non-intensive care [9, 10]. Acknowledging methodological constraints, the authors concluded that ICU admission represents good value for money and can compare with other essential health care services. Few cost-effectiveness studies of ICU admission take into account important individual variability in terms of the patients’ death risks, age, treatment needs and resource use, and potential survival benefit of admission [11, 21–24].

Rationing intensive care is inevitable and should follow explicit criteria such as cost-effectiveness [25, 26]. Cost-effectiveness must be balanced against other concerns that may conflict with or are not always captured by the use of CEAs—e.g. “rule of rescue”—the imperative to save patients from imminent death, respect for autonomy, or disease-related severity [14, 27, 28]. Expected lifetime health can be used to compare severity across diseases and individuals and is discussed as an explicit criterion for priority setting in Norway and endorsed by the World Health Organization under the name of priority to the worse off [13, 29]. A high risk of imminent death, profound pain, functional deterioration or reduced health-related quality of life, early rather than late onset of illness in life, and long duration of illness or disability will each, or together, contribute to a high degree of severity of disease. The ethical rationale is that patients with higher severity of disease deserve extra priority; improving their situation may reduce inequalities in expected lifetime health between patient groups with different conditions [30, 31]. White et al. proposed that a similar lifecycle argument could guide priorities in life support during a public emergency [32]. An explicit concern for severity of disease is rarely integrated into CEAs [33].

A substantial increase in ICU capacity may be necessary in the coming decades because of increased demand due to an ageing population and changing disease patterns [7]. Since CEA is increasingly used to judge the value of different types of health care (predominantly new interventions), we sought to evaluate the opportunity cost of ICU admission compared with some next-best alternative. It is impossible to remove confounding from such comparisons since we do not have good data from clinical trials. This modelling exercise necessarily requires a number of general assumptions about the differences in short- and long-term mortality, health-related quality of life, and resource use between those admitted or hypothetically not admitted to the ICU.

The aims of this paper are to describe the extra costs and expected health gains associated with admission to the ICU versus the general ward for 30,712 patients, and the variation in cost-effectiveness estimates among subgroups and individuals, and to perform a distribution-weighted economic evaluation incorporating extra weighting to patients with high severity of disease.

## Methods

### Conventional cost-effectiveness analysis

We developed a micro-simulation model to estimate the expected incremental cost in Euro (€) per quality-adjusted life year (QALY) gained from ICU admission for each individual ICU patient compared to hypothetical ICU rejection (resembling general ward care) of the same patient. The second best treatment option in our setting would be a regular hospital ward unequipped to provide highly specialized organ support. Intermediate care units are uncommon. The Norwegian Intensive Care Registry (NIR) is a good source of data for a model aiming to describe the variability of the cost-effectiveness of ICU admissions because it contains individual-level data on age, the length of the ICU stay, and death risk. Information about the study population, setting, and key model assumptions and parameters are included in Table 1. We updated a previously published model predicting the life expectancy of NIR patients from 2008 to 2010 (*n* = 40,916) [34]. Patients with missing data, Simplified Acute Physiology Score (SAPS) II <1, ICU re-admission during the same hospital stay, or transfers to other hospitals were excluded. A total of 30,712 ICU patients were included in this study. Details of the excluded patients were reported previously [34]. Table 2 includes baseline characteristics of the study population. We used input from secondary sources when needed. The literature search is shown in Additional file 1.

**Table 1:** Key model assumptions and parameters

Key model assumptions		Description			Sources
Patients		General adult ICU population (*n* = 40,916), Norway 2008–2010. One or more organ failures, case mix, >90% of annual ICU admissions in the country			Norwegian Intensive Care Registry
Intervention		ICU admission			
Comparator		Hypothetical ICU refusal and general ward treatment of the same patients			
Setting		38 mixed medical-surgical ICUs out of a total of 42 ICUs in publicly funded hospitals operated by local health trusts under four state-owned regional health authorities.			
Health outcome		Quality-adjusted life years (QALYs)			
Resource use		Hospital costs were summed in Norwegian kroner (NOK), converted to NOK in 2016 using the CPI and to Euro (€) using OECD PPP: 1 USD = 9.4 NOK = 0.75 Euros			Statistics Norway, [51]
Severity of disease		Expected lifetime health if rejected to the ICU. Measured as number of lifetime QALYs.			[13]
Distribution weights		Higher weights assigned to health gains to patients with more severe conditions; results in more favourable cost-effectiveness estimates.			Additional file 2, [13, 62]
Perspective		Hospital, health care provider			[50]
Time horizon		Lifetime			[50]
Discounting		4% annually of health benefits and incremental health care costs post-hospital discharge.			[50]
Heterogeneity		Analysis of total study population, subgroups by type of admission and individual admissions.			
General cost-effectiveness threshold range		275,000–800,000 NOK per QALY ≈22,000–64,000 PPP-adjusted € per QALY.			[56, 57]
Model parameter		Parameter values			Sources
.		Base case	Range	Distribution	.
Age		Individual age	Individual age	Observed	ICU cohort 2008–2010
Transition probabilities:					
Effect of ICU admission on hospital mortality					
Predicted hospital mortality if ICU patient		Individual SAPS II, calibrated model		Observed	ICU cohort 2008–2010, [35]
Predicted hospital mortality if general ward		Individual SAPS II, modified model (*β1* = 0.09)	*β1* = 0.0737 to 0.14	Scaled beta^a^	Fig. 1, Additional files 1 and 2
Annual mortality after hospital discharge		Age-specific *3–1 over 3 years	*1 to *5–1 over 10 years	Uniform	Life table Norway 2011, [34], Additional file 2
Length of hospital stay, mean (median) days					
If ICU, weighted sum of					
Died ICU	LOS ICU	4.8 (1.7)	0.6–5.0 (IQR)	Observed	ICU cohort 2008–2010
	LOS ward post ICU	0	NA	NA	
Died ward	LOS ICU	5.4 (2.6)	1.3–5.9 (IQR)	Observed	ICU cohort 2008–2010
	LOS ward post ICU	2.5	NA	Gamma (scale = 0.5, shape = 5)	
Survived hospital	LOS ICU	4.1 (2.0)	1.1–4.1 (IQR)	Observed	ICU cohort 2008–2010
	LOS ward post ICU	10	NA	Gamma (scale = 2, shape = 5)	
If general ward, weighted sum of					
Died hospital	LOS ward	LOS if ICU and dead *0.9	NA	Gamma	
Survived hospital	LOS ward	LOS if ICU and hospital survivor *1.1	NA	Gamma	
Cost units					
Cost of ICU bed-day		€3980	€2390 to €5570	Scaled beta^b^	Additional file 2, [18]
Cost of general ward bed-day		€640	€320 to €950	Scaled beta^b^	Additional file 2
Annual health care cost survivors, year 1		€6400	€80 to €12,700	Scaled beta^b^	[47, 48]
Years 2–5		Year 1 *0.6, *0.5, *0.4, *0.3			
Health-related quality of life weights		*0.8	*0.6 to 0.9	Scaled beta^c^	Additional files 1 and 2, [19, 38–42]
Age-matched reference value reduced 20% for 5 years after discharge, equally for both treatment options	18–19: 0.90	0.72	0.54 to 0.81		
	20–29: 0.89	0.71	0.53 to 0.80		
	30–39: 0.88	0.70	0.53 to 0.79		
	40–49: 0.86	0.69	0.52 to 0.77		
	50–59: 0.84	0.67	0.50 to 0.76		
	60–69: 0.81	0.65	0.49 to 0.73		
	70–79: 0.79	0.63	0.47 to 0.71		
	80+: 0.73	0.59	0.44 to 0.66		
Distribution weights based on lifetime QALYs		Linear	NA	NA	Additional file 2, [13, 62]

Scaled beta: min + (max – min) × beta(alpha,beta)

^a^beta(alpha = 2,beta = 3.5)

^b^beta(alpha = 2,beta = 2)

^c^beta(alpha = 4,beta = 2

*CPI* consumer price index, *ICU* intensive care unit, *IQR* interquartile range, *LOS* length of stay, *NA* not applicable, *NOK* Norwegian kroner, *PPP* purchasing power parity, *SAPS* Simplified Acute Physiology Score, *USD* United States dollar

**Table 2:** Baseline characteristics of the study population

Patient characteristic	All	Medical	Acute surgery	Planned surgery
*n*	30,712	17,122	9722	3868
Age (years)				
Mean (SD)	63.2 (18.2)	63.7 (18)	61.4 (19.3)	65.2 (15.4)
Q1	52.4	53.4	48	57.2
Median	66	66.5	64.7	67.5
Q3	77.3	77.7	77	76.5
SAPS II				
Mean (SD)	36.8 (18.2)	38.6 (18.8)	36.9 (17.2)	29 (16)
Q1	24	25	24.2	18
Median	34	36	35	25
Q3	47	49	47	37
Survival status (proportion)				
Died ICU	0.13	0.15	0.12	0.05
Died ward	0.07	0.07	0.06	0.05
Survived hospital	0.81	0.78	0.82	0.9
LOS ICU (days)				
Mean (SD)	4.3 (6.8)	4 (6.4)	5 (7.5)	3.8 (6.6)
Q1	1.1	1	1.3	1.1
Median	2	1.9	2.3	1.9
Q3	4.3	4	5.2	3.4

*ICU* intensive care unit, *LOS* length of stay, *Q* quartile, *SAPS* Simplified Acute Physiology Score, *SD* standard deviation

For each treatment alternative in the micro-simulation, a Markov process was run separately for 30,712 individuals to account for the variability of individual model input values represented by their unique profiles of predicted short-term risk of death based on SAPS II, age, and length of ICU stay obtained from NIR [35]. In probabilistic sensitivity analysis, we ran the cohort of all individuals 1000 times and sampled from the range of possible parameters for each mean value. The uncertainty around each variable is based on reported cost data or previous studies (modified SAPS II model, health-related quality of life (HRQoL) weights, long-term mortality, total length of hospital stay, cost of ICU and general ward bed day, mean annual health care cost 5 years after initial hospital discharge) [36].

### Health gain from ICU admission

**Fig. 1:**
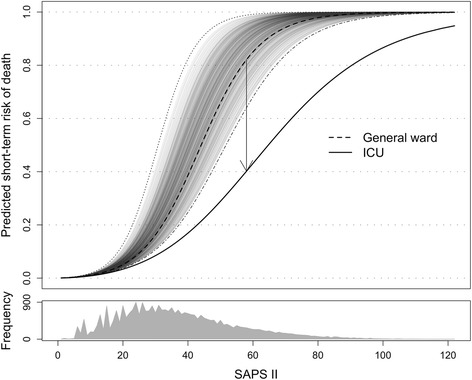
Short-term survival benefit of admission to the ICU versus the general ward: assumptions. SAPS II versus risk of death if admitted to the intensive care unit (*ICU*) or general ward (base case). *Multiple grey lines* represent the range of modified SAPS II models used in the analysis of uncertainty. For a given SAPS II, the vertical distance between the two lines represents the absolute short-term survival benefit of ICU admission compared to general ward care. For example, a sepsis patient with a predicted hospital mortality of 40% with treatment in the ICU would be attributed an absolute mortality reduction of 42% from admission (indicated by *arrow*, corresponding relative risk ratio = 0.49). The mountain-like *grey shape* at the bottom shows the distribution of patients according to SAPS II

We assumed the benefits of ICU admission versus ward care as displayed in Fig. 1 where the absolute short-term survival benefit (Fig. 1 and Additional file 2) [21, 37]: a) increases with SAPS II, and indirectly with age since SAPS II is age-weighted; b) peaks around mid- to high-range SAPS II; c) diminishes in each end of the SAPS II range, i.e. if too well or too sick to benefit, respectively; and d) can differ between patients too well and too sick to benefit, i.e. at each end of the SAPS II range.

The assumptions were based on a systematic search and rapid review of studies estimating the mortality impact of ICU admission compared with refusal (Additional files 1 and 2). The lower limit of the benefit assumption was based on the original SAPS II model published in 1993 which implies that admitting critically ill to general wards today would have about the same hospital mortality as admitting these patients to ICUs in the late 1980s (Fig. 1 and Additional file 2).

Subsequent survival after hospital discharge was estimated using the same life tables for both treatment options, corrected for excess mortality in ICU survivors, and adjusted by age-specific HRQoL weights from the general population. The HRQoL was down-weighted by 20% over the first 5 years after the hospital stay because we assumed that the HRQoL of ICU survivors persists at a lower level than the general population (Table 1 and Additional file 2) [19, 38–42].

### Resource use

We used a top-down costing method. The estimated cost of an ICU admission took into account both the cost of the initial hospital stay and resource use among survivors up to 5 years after discharge. Similar estimates were obtained for patients if treated in the general ward. The extra cost of ICU admission versus ward care for an individual patient was the difference between the estimated total ICU and ward costs, given the patient’s characteristics.

The cost per day in the ICU is highest in the first 24 h and then falls substantially [4, 43]. Normalised to the average cost of an ICU bed-day, we modelled ICU daily costs such that ICU days 1 and 2 were 3- and 1.5-times costlier, respectively, than ICU day 3 onwards. The ward stay cost post-ICU was estimated by multiplying the length of stay with the average cost of a general ward bed-day. We used distributions of the average cost of an ICU bed-day, €3980 (range €2390 to €5570), and the average cost of a general ward bed-day, €640 (range €320 to €950), based on data emerging from hospitals piloting a cost-per-patient specification issued by The Norwegian Directorate of Health (Table 1, see also Additional file 2) [44].

If the patient was not admitted to the ICU but treated in the general ward, we modelled the daily cost over the course of an individual’s estimated length of hospital stay. Assuming that these patients would demand extra resources in the ward, the first days were weighted differentially as described above, but normalised to the average cost of a general ward bed-day, €640 (range €320 to €950) (see Additional file 2).

NIR provides an individual’s observed length of the ICU stay (LOS ICU). For a patient admitted to the ICU, we calculated the expected LOS ICU as the weighted sum of sampled LOS of individuals of the same age group (age ±5 years) and the same type of admission from three categories in NIR: those who died in the ICU (66% of the non-survivors), died in the ward (34% of the non-survivors), or survived hospital (weighted by the patient’s probability of dying or surviving the hospital stay given by calibrated SAPS II; Tables 1 and 2). The expected number of ward days following ICU discharge (LOS ward) was estimated based on available references from Norway and a European multi-centre study of ICU triage [37, 45, 46]. For ICU decedents, we set LOS ward to 0 days, and for those who died in the ward after ICU discharge we sampled LOS ward from a distribution of possible values (mean 2.5 days). For hospital survivors, LOS ward was sampled from a different distribution with a mean of 10 days. Then the weighted sum was calculated (Table 1).

The expected total hospital LOS for a patient being admitted to the general ward was based on what their total hospital LOS would have been if admitted to the ICU. If the patient would have died in the ICU, the total hospital LOS was reduced by 10% on average. If the patient would have survived the hospital stay, the total hospital LOS was increased by 10% on average. Then the weighted sum was calculated based on the patient’s probability of dying or surviving the hospital stay in the case of general ward treatment (given by modified SAPS II; Tables 1 and 2). These assumptions allow for a patient treated in the general ward to have a longer or shorter hospital stay than if admitted to the ICU.

Survivors of critical illness demand substantial health care resources in the years following hospital discharge [47]. Data on long-term health care resource use among survivors from the general ICU population is lacking in Norway. In a recent study from Scotland, Lone et al. found that over a 5-year follow-up period more than 80% of an ICU cohort had at least one hospital admission, with a mean of 4.8 admissions [48]. They reported mean annual hospital costs per years 1 to 5, which we included in our model with broad ranges around the mean values. We assumed that long-term hospital costs were equal between survivors of the two treatment options, and calculated the weighted sum for each patient.

Norwegian guidelines for health economic evaluations recommend that we use cost per QALY analysis and that a 4% annual discount rate is applied to both health benefits and costs. A health care provider perspective is used here [49, 50]. Costs were converted from Norwegian kroner (NOK) to Euros (€) using OECD purchasing power parities [51].

### Distribution-weighted economic evaluation

We explored a method to incorporate a concern for severity of disease into CEAs by assigning higher weights to health gains for patients with less expected lifetime health without the intervention in question [14]. Lifetime QALYs have been suggested as a measure that can be used to compare severity across diseases and individuals [13, 52, 62] . For a patient referred to the ICU, the lifetime QALYs were calculated by the sum of a) past years lived with a quality of life as the general population at specific ages and b) the future QALYs given the increased risk of death and loss of quality of life due to acute illness or trauma if rejected to the ICU. In this model, severity of disease depends on short- and long-term risk of death, age, and HRQoL in the past and future. Additional file 2 provides details of the weighting function.

### Scenario analyses

In one scenario analysis, we applied an almost constant ICU or ward daily cost (days 1, 2, and 3 were 1.2-, 1.1-, and 1.05-times costlier, respectively, than day 4 onwards), acknowledging that the variation in the cost from one day to another may not be as substantial as reported elsewhere due to high staffing (nurse:patient ratio), short mean and median ICU stays, and a low number of ICU beds to hospital beds and population size (Table 2) [5]. In another scenario analysis, we accounted for lifetime health care costs beyond 5 years among hospital survivors, as recommended by US general and ICU guidelines for CEAs, to improve the generalizability of our findings [27, 53]. Out of NOK 277 billion public spending on health care in 2016, about 50% (€2070 per capita) goes to curative care mainly in hospitals, and about 30% (€1190 per capita) to long-term care in facilities or at home [54]. We added the average annual health care cost (mean €2070, range ± €1190) from year 6 after the initial hospital stay (scaled beta distribution: €880 + (€3260 – €880) × beta(alpha = 2,beta = 2).

All calculations and analyses were performed using R programming [55]. A cost-effectiveness threshold range between €22,000 and €64,000/QALY was used as a reference. This is supported by a recent White Paper endorsed by Parliament, even if there is no officially approved cost-effectiveness threshold in Norway. Threshold levels vary by severity of disease, where severe diseases have higher thresholds and less severe diseases have lower thresholds. The minimum threshold should correspond to the opportunity cost in the health services, which has been estimated to be about NOK 275,000 (€22,000) [56]. Currently, the national Decision Forum approves reimbursement of new health technologies up to around NOK 800,000/QALY (€64,000/QALY) [57].

## Results

### Standard cost-effectiveness analysis

Mean results from the standard cost-effectiveness analysis based on aggregated individual results, i.e. the sum of extra costs divided by the sum of gains for all patients and by type of admission, are shown in Table 3. The cost-effectiveness of ICU admission versus ward care was €11,600/QALY, with 1.6 QALYs gained from ICU admission and an incremental cost of €18,700 per patient. Medical admissions (€10,700/QALY) had a more favourable incremental cost-effectiveness ratio (ICER) than admissions after acute surgery (€12,300/QALY) and after planned surgery (€14,700/QALY).

**Table 3:** Mean cost-effectiveness of ICU admission versus hypothetical general ward care. Data from Norway*

Patient group	ICU strategy	Costs	Incremental costs	QALYs	Incremental QALYs	Incremental C/E	Prob C/E^a^	Distr C/E^b^
All (*n* = 30,712)								
	Reject	16,100		6.1 (11.6)				
	Admit	34,800	18,700	7.7 (14.4)	1.6 (2.8)	11,600	0.95	5000
Medical (*n* = 17122)								
	Reject	15,300		5.7 (10.9)				
	Admit	33,500	18,200	7.4 (13.8)	1.7 (2.9)	10,700	0.97	4600
Acute surgery (*n* = 9722)								
	Reject	16,200		6.5 (12.9)				
	Admit	36,900	20,700	8.2 (15.8)	1.7 (2.9)	12,300	0.93	5400
Planned surgery (*n* = 3868)								
	Reject	19,200		6.6 (11.5)				
	Admit	35,400	16,200	7.7 (13.4)	1.1 (1.9)	14,700	0.84	6500

* The numbers are average extra costs in Euro or health gains in quality-adjusted life years (QALYs) per patient. Costs and QALYs were discounted at 4% annually (undiscounted QALYs in brackets)

^a^Using a general cost-effectiveness threshold of €22,000/QALY

^b^Results after health gains were weighted according to the patient’s lifetime QALYs in case of general ward care (severity of disease)

*C/E* cost-effectiveness, *Distr* distribution-weighted, *Prob* probability

**Fig. 2:**
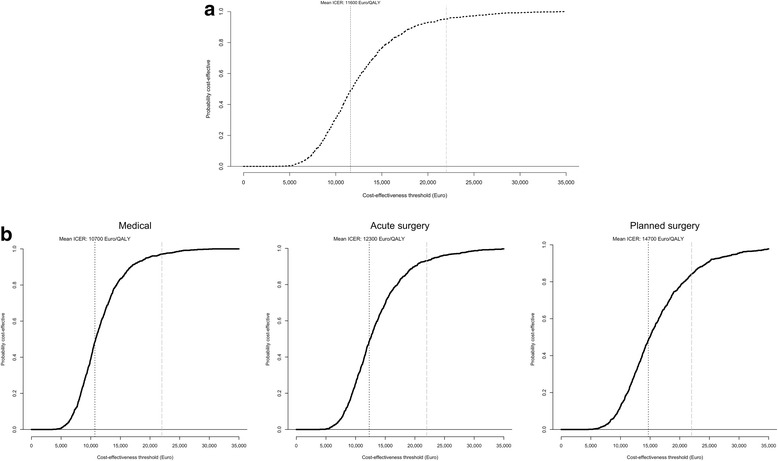
**a** Cost-effectiveness acceptability curve: all patients. The probability that ICU admission versus general ward care was cost-effective was 95% at a threshold of €22,000/QALY (threshold indicated by *long dashed line*). **b** Cost-effectiveness acceptability curve: by type of admission. The probability that ICU admission versus general ward care was cost-effective by type of admission. Threshold of €22,000/QALY indicated by *long dashed line*. ICER incremental cost-effectiveness ratio, QALY quality-adjusted life year

Figure 2 shows the cost-effectiveness acceptability curve based on mean ICERs from the analysis of uncertainty. From a cost-effectiveness perspective, the probability that ICU admission should be preferred to general ward care was 95% for all patients, 97% for medical, 93% for acute surgical, and 84% for planned surgical patients at a threshold of €22,000/QALY.

**Fig. 3:**
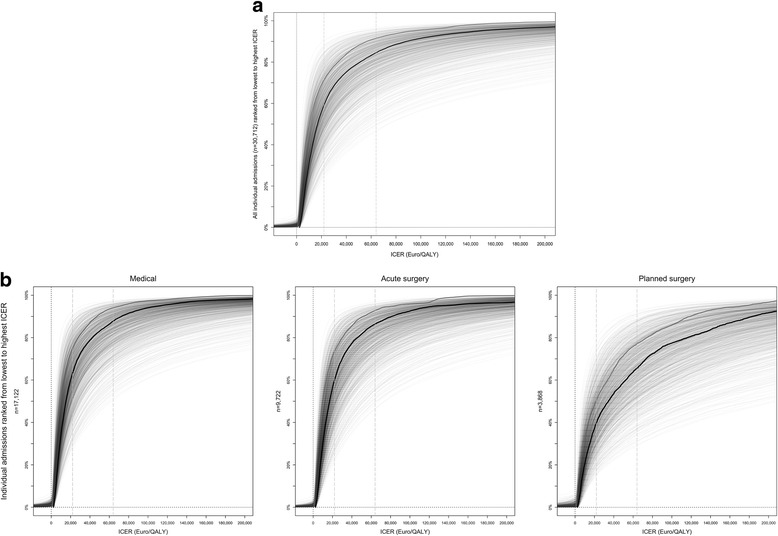
**a** Individualized cost-effectiveness with and without distribution weights for severity of disease: the disaggregated individual results. Each line is made up of 30,712 points. Each point represents the ICER for an individual admission. The individualized ICERs are sorted from the lowest (*left*) to the highest (*right*) ICER. There was a 50% probability (median, *black line*) that ICU admission was cost-effective for 85% of the patients at a threshold of €64,000/QALY (*long dashed line*). The figure illustrates that after assigning distribution weights according to severity of disease, i.e. higher weights to the health gains of patients with fewer lifetime QALYs if rejected, ICU admission can be considered acceptable for more patients (*thick grey line*) for any cost-effectiveness threshold compared to the standard analysis (*black line*). **b** Individualized cost-effectiveness in subgroups by type of admission. The individualized incremental cost-effectiveness ratios were plotted as points forming a line. The individualized ICERs are sorted from the lowest (*left*) to the highest (*right*) ICER. The *thick black line* is the median result for each individual from 1000 replications of the model. The *long dashed line* indicates a general cost-effectiveness threshold of €64,000/QALY. ICER incremental cost-effectiveness ratio, QALY quality-adjusted life year

Figure 3 shows the disaggregated individual results. Each line is made up of 30,712 points. Each point represents the ICER for an individual admission. The individualized ICERs are sorted from the lowest (left) to the highest (right) ICER. One line represents the variability across modelled individuals. The cloud of 1000 light grey lines represents parameter uncertainty.

The black line shows the median individualized results from the probabilistic analysis. The median is the equivalent to 50% probability of cost-effectiveness. There was a 50% probability (median) that ICU admission was cost-effective for 85% of the patients at a threshold of €64,000/QALY (Fig. 3a). The remaining 15% median individualized ICERs were above €64,000/QALY, and constituted 12% of medical admissions, 14% of admissions after acute surgery, and 34% of admissions after planned surgery (probability = 50%; Fig. 3b).

On average, ICU admission was more costly than general ward care for all draws in the probabilistic analysis. However, the lines left of zero on the *x* axis in Fig. 3a and b illustrate that ICU admission was found to be cost-saving in a small proportion of the individualized models.

### Distribution-weighted economic evaluation

The ICER for the entire intensive care population was €11,600/QALY; the impact of distribution weights according to severity of disease reduced this mean ICER to €5000/QALY. In the distributional evaluation, only 8% of all patients had distribution-weighted ICERs above €64,000/QALY due to higher weights to gains for more severe conditions (probability = 50%; Fig. 3). Patients with distribution-weighted ICERs above €64,000/QALY represented 6% of medical admissions, 7% of admissions after acute surgery, and 23% of admissions after planned surgery. All of these patients could expect more than 68 lifetime QALYs if not admitted to the ICU.

The average severity of disease was 66.5 undiscounted QALYs over a lifetime if not treated in the ICU, and the mean undiscounted gain from admission was 2.8 QALYs. Patients with the highest severity of disease (<50 lifetime QALYs, mean = 42.6 undiscounted QALYs) gained the most from admission, 13.8 undiscounted QALYs per patient on average, and these admissions were most cost-effective. Patients with low severity of disease (65+ lifetime QALYs, mean = 70 undiscounted QALYs) gained 1.1 undiscounted QALYs per patient. The resulting lifetime total if admitted to the ICU was 68.3 QALYs (all patients), 56.4 QALYs among patients with the highest severity of disease, and 71.1 among those with low severity of disease.

### Scenario analyses

In the scenario analyses the same pattern emerged as in the main analysis. The mean cost-effectiveness of ICU admission versus ward care was €12,000/QALY (*p* = 95%), with 1.6 QALYs gained from ICU admission and an incremental cost of €19,100 per patient, when assuming a smaller difference between the first and later daily costs. Medical admissions (€11,000/QALY, *p* = 98%) had a more favourable ICER than admissions after acute surgery (€13,000/QALY, *p* = 92%) and after planned surgery (€14,600/QALY, *p* = 84%). When we included lifetime health care cost, the mean cost-effectiveness of ICU admission versus ward care was €13,900/QALY (*p* = 91%), with 1.6 QALYs gained from ICU admission and an incremental cost of €22,200 per patient. Medical admissions (€13,000/QALY, *p* = 95%) had a more favourable ICER than admissions after acute surgery (€14,600/QALY, *p* = 87%) and after planned surgery (€17,100/QALY, *p* = 73%).

## Discussion

The main finding of this modelling study is that the average health gain per patient from ICU admission was 1.6 QALYs at an incremental cost of €18,700 compared with treating the same patients in general wards. The resulting mean ICER of ICU admission was €11,600/QALY, and would be considered cost-effective over common cost-effectiveness thresholds, including the Norwegian setting at a threshold range of €22,000–64,000/QALY. The probability of ICU admission being cost-effective was 95% at a threshold of €22,000/QALY. By contrast, the disaggregated results demonstrate a much greater uncertainty given by the range in probability of cost-effectiveness due to individual variability and uncertainty around input parameters. There was a 50% probability that ICU admission was cost-effective for 85% of the patients at a threshold of €64,000/QALY. At this level of uncertainty, about one sixth of the individual patient admissions were estimated to be not cost-effective. Half of these, and consequently 92% of all individual admissions, had an acceptable relationship between resource use and health gains in the distributional analysis giving extra weight to health gains for those with higher severity of disease (probability = 50%).

Our study contributes in three main areas. First, it is the first study from a Nordic country that estimated the difference in costs and QALYs between ICU admission and ward care. Second, we account for the heterogeneity and distribution of incremental costs and health gains in a nationwide ICU population. We modelled individual admissions using real data from NIR to describe variability across admissions; the variation in the cost-effectiveness across individuals is due to observed differences in their profile of length of stay, short-term risk of death and age, and effect size. Third, in the explorative distribution-weighted evaluation we adjusted the ICER by severity of disease in terms of lifetime QALYs. ICU admission was more cost-effective among high-severity patients (lifetime QALYs <50). Our study is among the first to incorporate a concern for severity of disease into a standard cost-effectiveness model by giving higher weight to health gains for the critically ill with fewer lifetime QALYs if not admitted [58, 62]. This approach is relevant to health care systems where priority setting aims at the twin goals of maximizing healthy life years for all and reducing inequalities in lifetime health across individuals or patient groups [13].

The main conclusion of the standard CEA is similar to other studies from Europe that sought to estimate the difference in costs and health outcomes between adult patients accepted and rejected to the ICU. The mean extra costs of ICU versus non-ICU care per patient of €18,700 are higher than previous reports. Edbrooke et al. estimated the extra hospital costs to be €4886 in 2005 (multicentre, Europe) [9]. Ridley and Morris included extra hospital costs plus discounted lifetime average health care costs of extra survivors due to ICU support (£8902 in 2003, UK) [10]. NIR lacks data about the long-term economic consequences of critical illness. We therefore applied available data from Scotland on hospital resource use in ICU survivors up to 5 years after the initial admission [48]. ICU admission after planned surgery was less cost-effective than after acute surgery and medical admissions. This can be explained by a lower hospital mortality, lower SAPS II and, therefore, lower expected health benefits (1.1 QALYs) from ICU admission after planned surgery (Tables 2 and 3). Edbrooke et al. found that patients with low, medium, and high predicted short-term risk of death have variable effect from ICU admission, and concluded that ICU admission is more cost-effective with increasing severity as measured by SAPS II [9]. One strength of our study is that we varied the survival benefit according to SAPS II and estimated expected outcomes for individual admissions. The distribution of individualized ICERs shows that behind the average results for the population as a whole there are a number of patients where ICU admission is predicted not to be cost-effective. In the probabilistic analysis, 15% of the patient profiles had a median cost to effect ratio of more than €64,000/QALY. Currently, new health care technologies such as cancer immunotherapies with an ICER above this limit will not be reimbursed. Prioritising interventions with such high ICERs may displace existing health care activities that benefit patients more.

We note some limitations of our analysis. We rely on crude estimates of the incremental costs of ICU versus ward care. We used the perspective of a decision maker allocating resources within the hospital sector. We tried to capture differences in direct costs of care for and health benefits to the critically ill patients, but acknowledge that there might be differences in the quality of end-of-life care and in the burden of critical care and illness felt by patients and family members in the ICU compared with the ward group [27]. The assumptions regarding the hospital length of stay and costs for both treatment options may under- or overestimate the difference in hospital costs. The length of the hospital stay was unavailable in NIR. The probability that ICU admission was cost-effective at a threshold of €22,000/QALY was high in the main analysis and scenario analyses, despite the uncertainty around possible input parameters and the broad ranges used. In scenario analysis, the extra costs of ICU admission increased for medical admissions (€18,400) and after acute surgery (€21,500) when we reduced the differences in daily costs throughout the ICU or ward stay. The extra cost decreased in admissions after planned surgery where ICU LOS was shorter compared with the other types of admission (€15,700; Table 2). A major challenge is the hypothetical nature of the comparator. Since RCT data are not available, all analysis must rely on counterfactual assumptions. The assumptions about the effect of ICU admission were informed by observational studies where patients refused and accepted to the ICU are likely to differ in case mix. We suspect effect estimates from these studies would underestimate the mortality reduction among those who benefit the most from ICU admission in a nationwide ICU population. We sought to compensate for lack of robust effect data by assuming that those with mid- to high-range SAPS II benefit the most, as illustrated in Fig. 1 [21]. Our study does not capture well how hospitals deal with levels of care below high-level ICU (multi-organ support), but above a regular ward. However, the issue is partly taken into account through the uncertainty range of model parameters (see Additional file 2 or effect and costs assumptions). The adjustment for excess long-term mortality and reduced HRQoL in hospital survivors could be stratified by patient categories (acute respiratory distress syndrome (ARDS), post-sepsis, cancer, etc.). We did not have such information to classify the NIR patients, and do not know if making more detailed distinctions among patients would impact on overall results or the distribution of individualized ICERs.

The methodology using a large individual-level (ICU) database to account for sub-group heterogeneity and individual variability of costs and expected outcomes is relevant to any country considering cost-effectiveness information in priority setting to improve health as much as possible. The cost-effectiveness results may at least be generalizable to other Scandinavian countries as the case mix of our ICU populations and ICU and health systems organisation are similar [59]. The study provides useful contextual information about the relationship between extra costs and health gains associated with current ICU care compared to other high-cost health care competing for the same resources [60]. Introducing new high-cost technologies with less favourable cost-effectiveness ratios than ICUs may have opportunity costs in terms of healthy life years forgone. The study does not directly inform a priority-setting task of choosing between specific future policy options since it does not assess a new intervention, such as scaling up ICU care by admitting more or other types of patients from the at-risk population before triage, or modernizing care of the critically ill by introducing intermediate care units [3, 17].

Even if patient groups may be denied access to an intervention because the mean group-level cost-effectiveness ratio of the intervention is above an accepted threshold value, clinicians may identify individual cases who are likely to benefit more and/or cost less than the expected average [61]. In intensive care, our findings suggest that we have the opposite case: overall ICU admission versus ward care is predicted to be cost-effective, but a number of patients are expected to have ICU admissions that are not cost-effective. If we can identify these types of individual ICU patients, should we then deny them treatment on grounds of cost-effectiveness? We believe drawing such a conclusion is premature. Given the difficulty of ICU triage and the uncertainty around selecting those who can benefit the most from admission to a reasonable cost, we may have to accept the great variability in individualized ICERs, and leave judgement to clinicians and clinical guidelines, as long as the average results can be considered cost-effective. Clinicians must be cautious about the assumptions underlying the individualized cost-effectiveness estimates and their use for a particular individual [11].

## Conclusion

This micro-simulation modelling study predicts that ICU admission versus general ward care of the same patients if not admitted to the ICU is likely to be cost-effective (mean ICER €11,600/QALY, *p* = 0.95) at a threshold of €22,000/QALY. The study provides a rough sense of the relationship between the expected costs of, and QALY gains from, ICU admission, and demonstrates that behind overall mean cost-effectiveness results there will be a great degree of variation and uncertainty of ICERs among individual types of ICU patients. According to the individualized cost-effectiveness information, about one in six ICU admissions are predicted to be not cost-effective at a threshold of €64,000/QALY. According to the distribution-weighted analysis, almost half of these not cost-effective admissions can be regarded as acceptable when weighted by severity of disease in terms of expected lifetime health. The analysis informs a public policy to expand ICU capacity to maintain today’s level of ICU services in the face of increased need due to changing demographics.

## Additional files

Additional file 1: Literature search. Strategies and results. (PDF 233 kb)

Additional file 2: Key model assumptions. Adjusted long-term survival, health-related quality of life, effect of ICU admission on short-term mortality, costs, distribution-sensitive weighting function. (PDF 1379 kb)

Additional file 3: Data file. Norwegian ICU cohort, 2008–2010. (CSV 1472 kb)

Additional file 4: Lifetable Norway, 2011. (XLSX 18 kb)

## Abbreviations

CEA: Cost-effectiveness analysis
HRQoL: Health-related quality of life
ICER: Incremental cost-effectiveness ratio
ICU: Intensive care unit
LOS: Length of stay
NIR: Norwegian Intensive Care Registry
NOK: Norwegian kroner
QALY: Quality-adjusted life year
RCT: Randomized controlled trial
SAPS: Simplified Acute Physiology Score
