# Supplementary material for: Costs and expected gain in lifetime health from intensive care versus general ward care of 30,712 individual patients: a distribution-weighted cost-effectiveness analysis
Source: Crit Care. 2017 Aug 21;21:220. doi: 10.1186/s13054-017-1792-0 (PMC5567919; doi:10.1186/s13054-017-1792-0)
Supplement: Supplementary file 1 — Literature search. Strategies and results. (PDF 233 kb) [file 13054_2017_1792_MOESM1_ESM.pdf]

Addtitional file 1

General search strategy:

We developed the general and specific search strategies from a knowledge base of ten publications [1-10]. With the objective to update the previous model with effect and cost assumptions and quality-adjustment weights [11], we first

1) searched for previous cost-effectiveness analyses (CEAs) and reviews of the effectiveness of intensive care using selected databases (Tufts CEA registry, - 2013, Cochrane library 2005-16 and Pubmed Health); then

2) searched for single studies of the benefit of ICU admission vs. non-intensive care from the year 2011 using Pubmed (Medline); then

3) performed a citiation search (Web of Science, 2006 -) for the articles in the knowledge base and those included from Pubmed results; and finally

4) went through the references in the articles that were selected in steps 1)-3).

We assessed retrievals in English (title, abstracts, or full text articles) according to their relevance to the PICO and exclusion criteria below. The quality of the studies was not assessed systematically. Lindemark performed the literature search and assessment.

PICO	Characteristics
**P**atients	Referred to the ICU. General ICU patients. Patients in intensive care or critical care wards. Adults, age 18+.
**I**ntervention	ICU admission. Intensive care. Critical care. ICU triage. ICU gatekeeping. Decision to admit to the ICU.
**C**omparator	Non-intensive care. General ward care. Refused, denied or delayed ICU care for any reason (too sick or too well to benefit, full bed-occupancy, other)
**O**utcome	Mortality, short- or long-term. Life years. Quality-adjusted life years.

**Exclusion criteria:**
Studies
- from which no survival benefit from ICU admission is reported/can be estimated
- reporting only ICU mortality
- of specific interventions within intensive care, e.g. early goal-directed therapy for sepsis
- of narrowly selected groups of ICU patients, e.g. neurosurgical patients, only old age
- of patients with age under 18
- assuming that patients die if not admitted to the ICU
- using ”do nothing” as comparator to ICU admission

The results of the systematic search for studies are shown in Table 1. The results of the search for utilities from the Tufts registry are shown in Table 2.

Table S1: Results of systematic search based on PICO

Source	Article type/database	n retrievals	n excluded**	Fulltexts evaluated	Included	Objective/Intervention	Comparator	Remarks
Tufts registry of CEAs	CEAs (updated through 2013)	Intensive care: 54	46	Linko CC 2010	No	MV for acute respiratory failure	None	Patient-level data. Describe average cost per QALY.
				Karlsson CCM 2009	No	Intensive care, severe sepsis	None	Patient-level data. Model. Finland. Describe average cost per QALY.
				Graf CC 2008	No	ICU adm after cardiac arrest	None	Patient-level data. Model. Germany. Describe average cost per QALY
				Huang CCM 2007	No	Goal-directed, sepsis		
				Ridley Anaesthesia 2007	Yes	ICU admission adults	General ward care	Hypothetical cohort, Uk, model. Review of effectiveness of ICU admission, Sinuff 2004 + three more articles
				Graf CCM 2005	No	Intensive care, medical admissions	Do nothing/No ICU care = death	Patient-level data. Model. Germany. Report cost per QALY.
		Critical care: 14	13	Hutchings BMJ 2009	No	Modernisation of adult intensive care		
		ICU admission: 1	1	0				
		Intensive care unit: 39	38	Talmor CCM 2008	No	Integrated sepsis protocol		
		Triage: 21	21	0				
Cochrane library	CDSR (issue 2, Feb 2016)	21	21	0				
	DARE (issue 2, April 2015*)	85	85	0				
	CCRCT (issue 1, Jan 2016)	481	481	0				
	HTA database (issue 1, Jan 2016)	3	3	0				HTA on ICU gatekeeping to be finalised 2018 by NIHR, UK
	NHS EED (issue 2, Apr 2015*)	71	70	Edbrooke CC 2011	Yes	ICU admission	ICU rejection, general ward	Based on patient data from ELDICUS study
PubMed Health		669	669	0				
PubMed	All	1083	1076	Louriz ICM 2012	Yes	ICU admission	Delayed ICU admision	
				Robert 2012 Am J R CCM	Yes	ICU admission	Delayed ICU admision	
				Sprung CCM 2012	Yes	ICU admission	ICU admission rejected	ELDICUS study, part II. Multi-centre, Europe.
				Howe AAIC 2011	No			n very low
				O'Callaghan CC 2012	No	ICU admission	Delayed ICU admission	Special case with boarding in theatre suite while waiting for avalable ICU bed
				Cardoso CC 2011	No	ICU admission	Delayed ICU admision	ICU mortality
				Simchen CCM 2007	No	ICU admission	Other ward care	Newly deteriorating patients who may be elibible to ICU. Specific a priori triage system.
Web of Science	All, citation search			Iapichino ICM 2010	No	ICU admission	ICU admission rejected	ELDICUS project
				Boumendil Plos One 2012	No	Analysis of variability of ICU admission		Age 80+
				Orsini JOCMR 2014	No			Only overall hospital mortality
Reference review	All			Garrouste-Orgeas ICM 2006	No			Age 80+
				Garrouste-Orgeas CCM 2009	No			Age 80+
				Shmueli IJTAHC 2005	Yes	ICU admission	ICU refusal	Hospital survival benefit and APACHE II score
				Sznajder ICM 2001	No	ICU stay (81.5% medical)	Do nothing/No ICU care = death	Report cost per QALY.
				Kerridge Anae. IC 1995	No	Analysis of QALYs in intensive care		

* Include publications up to March 2015

** Excluded based on title or abstract

Table S1: Results of search for utilities among ICU patients and survivors in CEA registry

Database	Retrievals	Author, journal year	Health state	Utility
Tuft CEA registry	1 intensive care unit: 95	Lehmann CC 2010	ICU sepsis	0,68
		Linko CC 2010	One year after ICU	0,7
		Cuthbertson BMJ 2009	One year Post ICU discharge	
			std care/with intervention	0.6/0.58
			6 months post ICU discharge	
			std care/with intervention	0.62/0.63
		Halton CC 2009	ICU patients 80 years +	0,66
			70-79 years old	0,75
			60-69 years old	0,79
			50-59 years old	0,8
			ICU admission	0,66
		Graf CC 2008	5 years post cardiac arrest and ICU survival, women	0.87
			5 years post cardiac arrest and ICU survival, men	0.74
		Graf CCM 2005	Survival after ICU stay	0,88
		MacLaren ValueHealth 2005	Post ICU health state	0,62
		Davies Anaesthesia 2005	Survivors of severe sepsis	0,69
		Sznajder ICM 2001	6 months after stay in ICU for:	
			SAPS II<50	0,52
			SAPS 35<50	0,57
			SAPS 20<35	0,68
			SAPS <20	0,64
			Acute surgery admission	0,7
			Elective surgery admission	0,71
			Medical admission	0,61
			Age > 65	0,63
			Age 40-64	0,58
			Age < 40	0,93
			Average self-assessment	0,63
		Kerridge ANAE IC 1995	Post-treatment for APACHE II 0-35+	0.96-0.85
	2 critical care, 27	Sud AmJRespC 2011	Former ICU patients discharged from hospital to home or nursing home	0,68
		Edwards 2012	UK patients recovering on the ward after discharge from critical care	
			UK patients in critical care ; best case	0,726317
			UK patients in critical care ; best case	0,598
	3 intensive care: 131	Ridley Anaesthesia 2007	Survived ICU	0,66
		Green IntJTAHC	Survivors of severe sepsis	0,6
	4 ICU admission: 1			

Specific search strategies:

CEA registry

Basic search Dec 9 2015. Retrievals through 2013.

For cost-effectiveness analyses:

Search terms (only new retrievals was added when using the following search term).

1 intensive care
2 critical care
3 ICU admission
4 intensive care unit
5 triage

For utilities (health-related quality of life weights):

Search terms (only new retrievals were added when using the following search term).

1 intensive care unit
2 critical care
3 intensive care
4 ICU admission

Studies were recorded if the health states were relevant to patients in or after ICU.

Cochrane library, all databases: 661 retrievals

Search Name:	ICU admission MESH search
Last Saved:	26.02.16 09:24
ID	Search
#1	MeSH descriptor: [Critical Care] explode all trees
#2	MeSH descriptor: [Intensive Care Units] explode all trees
#3	#1 or #2
#4	MeSH descriptor: [Referral and Consultation] explode all trees
#5	MeSH descriptor: [Decision Making] explode all trees
#6	MeSH descriptor: [Triage] explode all trees
#7	MeSH descriptor: [Patient Admission] explode all trees
#8	MeSH descriptor: [Health Care Rationing] explode all trees
#9	MeSH descriptor: [Health Priorities] explode all trees
#10	MeSH descriptor: [Resource Allocation] explode all trees
#11	MeSH descriptor: [Ethics] explode all trees
#12	MeSH descriptor: [Vital Statistics] explode all trees
#13	ration*
#14	refuse*
#15	deni*
#16	deny
#17	ICU admission
#18	triag*
#19	#4 or #5 or #6 or #7 or #8 or #9 or #10 or #11 or #12 or #13 or #14 or #15 or #16 or #17 or #18
#20	#3 and #19 Publication Year from 2005 to 2016

PubMed Health: 669 retrievals

March 1 2016

(((((("Critical Care"[Mesh]) OR "Intensive Care Units"[Mesh]))
AND
(((((((((((((("Referral and Consultation"[Mesh])) OR "Decision Making"[Mesh]) OR "Triage"[Mesh]) OR "Health Care Rationing"[Mesh]) OR "Health Priorities"[Mesh]) OR "Bioethics"[Mesh]) OR "Vital Statistics"[Mesh]) OR ration*) OR refuse*) OR deni*) OR deny) OR "ICU admission") OR triag*) OR benefi*)))
AND
((mortality) OR survival))

Pubmed (Medline): 1083 retrievals

Filters: publication date last 5 years March 1 2016 Time 07:05:19

"Search ((((intensive care[MeSH Terms]) OR intensive care unit[MeSH Terms]))
AND
((((((((refus*) OR deny) OR denied) OR triage[MeSH Terms]) OR health care rationing[MeSH Terms]) OR allocation, resource[MeSH Terms]) OR health priorities[MeSH Terms]) OR ""ICU admission""))
AND
mortality[MeSH Terms]"

Web of Science Core Collection

Citation search for articles in the knowledge base. Time: March 7 2016. Studies from last 10 years (2006 -)

Citation search for articles included from Pubmed results. Time: March 7 2016. Studies from last 10 years (2006 -)

1. Sinuff T, Kahnamoui K, Cook DJ, Luce JM, Levy MM, (2004) Rationing critical care beds: a systematic review. Crit Care Med 32: 1588-1597
2. Sprung CL, Geber D, Eidelman LA, Baras M, Pizov R, Nimrod A, Oppenheim A, Epstein L, Cotev S, (1999) Evaluation of triage decisions for intensive care admission. Crit Care Med 27: 1073-1079
3. Sprung CL, Artigas A, Kesecioglu J, Pezzi A, Wiis J, Pirracchio R, Baras M, Edbrooke DL, Pesenti A, Bakker J, Hargreaves C, Gurman G, Cohen SL, Lippert A, Payen D, Corbella D, Iapichino G, (2012) The Eldicus prospective, observational study of triage decision making in European intensive care units. Part II: intensive care benefit for the elderly. Crit Care Med 40: 132-138
4. Sprung CL, Baras M, Iapichino G, Kesecioglu J, Lippert A, Hargreaves C, Pezzi A, Pirracchio R, Edbrooke DL, Pesenti A, Bakker J, Gurman G, Cohen SL, Wiis J, Payen D, Artigas A, (2012) The Eldicus prospective, observational study of triage decision making in European intensive care units: part I--European Intensive Care Admission Triage Scores. Crit Care Med 40: 125-131

5. Sprung CL, Danis M, Iapichino G, Artigas A, Kesecioglu J, Moreno R, Lippert A, Curtis JR, Meale P, Cohen SL, Levy MM, Truog RD, (2013) Triage of intensive care patients: identifying agreement and controversy. Intensive Care Med 39: 1916-1924
6. Ridley S, Morris S, (2007) Cost effectiveness of adult intensive care in the UK (Structured abstract). Anaesthesia DOI
7. Talmor D, Shapiro N, Greenberg D, Stone PW, Neumann PJ, (2006) When is critical care medicine cost-effective? A systematic review of the cost-effectiveness literature. Crit Care Med 34: 2738-2747
8. Metcalfe MA, Sloggett A, McPherson K, (1997) Mortality among appropriately referred patients refused admission to intensive-care units. Lancet 350: 7-11
9. Garrouste-Orgeas M, Montuclard L, Timsit JF, Misset B, Christias M, Carlet J, (2003) Triaging patients to the ICU: a pilot study of factors influencing admission decisions and patient outcomes. Intensive Care Med 29: 774-781
10. Flaatten H, Kvale R, (2003) Cost of intensive care in a Norwegian University hospital 1997-1999. Crit Care 7: 72-78
11. Lindemark F, Haaland OA, Kvale R, Flaatten H, Johansson KA, (2015) Age, risk, and life expectancy in Norwegian intensive care: a registry-based population modelling study. PLoS One 10: e0125907
