# Supplementary material for: Costs and expected gain in lifetime health from intensive care versus general ward care of 30,712 individual patients: a distribution-weighted cost-effectiveness analysis
Source: Crit Care. 2017 Aug 21;21:220. doi: 10.1186/s13054-017-1792-0 (PMC5567919; doi:10.1186/s13054-017-1792-0)
Supplement: Supplementary file 2 — Key model assumptions. Adjusted long-term survival, health-related quality of life, effect of ICU admission on short-term mortality, costs, distribution-sensitive weighting function. (PDF 1379 kb) [file 13054_2017_1792_MOESM2_ESM.pdf]

Additional file 2

Model structure and assumptions:

Fig 4 Health state diagram

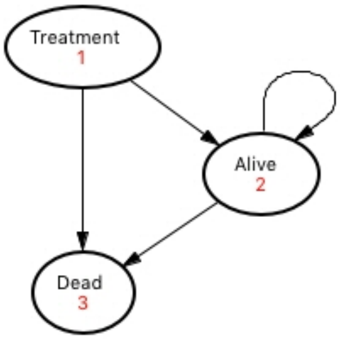

We used three states to describe the Markov process: Each patient started alive in a treatment health state, either as an ICU patient or as a hypothetical general ward patient, and ended up in a health state as either dead or alive. Transitions from the treatment state to death were calculated from the SAPS II for each individual using a recalibrated ("ICU") or modified ("General ward") SAPS II model [1]. See more about effect assumptions below.

Long-term survival:
We extrapolated mortality data of survivors of the hospital stay using age-specific annual death risks from a Norwegian life table. We adjusted the death risks to account for on-going excess mortality in ICU survivors compared to the general population. The details of this adjustment have been described previously [2]. We found Markov modelling appropriate due to the lifetime horizon and the need to model events of death for independent individuals at different ages. The table below illustrates how the adjustment of the age-specific annual probability of death (qx) influences the life expectancy (LE) at selected ages:

Lifetable	LE at 50	LE at 60	LE at 70	LE at 80	LE at 90
Norw. 2011 unadjusted, qx*1	33.79	24.78	16.53	9.47	4.58
qx*3-1 over 3 years	33.53	24.33	15.8	8.24	2.81
qx*5-1 over 10 years	32.09	21.82	12.17	4.24	0.94
qx*8-1 over 30 years	23.19	12.36	5.83	2.07	0.5

In probabilistic sensitivity analysis, we sampled randomly from three categories of life expectancies: qx*1 (no adjustment), qx*3-1 over three years, and qx*5-1 over 10 years.

Health-related quality of life:
We assigned the same age-specific health-related quality of life weights (HRQoL) to ICU survivors and counterfactual general ward survivors. The source of the HRQoL was a Swedish general population (mean 0.81). Reference HRQoL from the Norwegian general population is lacking. The HRQoL were down-weighted by 20% on average over the five first years after the hospital stay (mean 0,65) because we assumed that the HRQoL of ICU survivors persist at a lower level than the general population. This is supported by Norwegian studies and a more recent study from the UK, where Cuthbertson *et al.* found that a HRQoL of 0.66 persist across five years post ICU [3, 4]. In probabilistic sensitivity analysis, we sampled randomly from age-specific reference HRQoL values down-weighted by 10 to 40% (scaled beta distribution, mean 20%). In effect, the HRQoLs assigned to the modelled individuals largely cover the range of utilities from the Tufts registry listed in Additional file 1 (see also Table 1 in main article).

Effect of ICU admission on short-term mortality: Rapid review of studies
Building on information about the short-term survival benefit from ICU admission vs. refusal reported in the Eldcus II study published in 2012 and the systematic reviews by Ridley *et al.*, Talmor *et al.* and Sinuff *et al.*, we did a systematic search for reviews and single studies [5-8]. See Appendix 1 for search strategies, databases, and results. We identified two additional studies that reported estimates of the short-term survival benefit from ICU admission [9, 10].

Randomised controlled trials that study the effect of ICU admission are lacking on ethical grounds. Studies that sought to estimate the effect of ICU admission vs. some next best alternative, such as general ward treatment, have taken the form of retro- or prospective analysis of patients referred to an ICU and compared the outcome of those admitted to those not admitted (Table 1). Several factors make the estimates of the survival benefit from admission hard to compare across studies:

a) different time to follow-up (ICU, hospital, 28- or 30-day)
b) different reasons for refusal (too well to benefit, too sick to benefit, full ICU, other reason) and variable reporting of average or separated effect data for these categories
c) different treatment alternatives for refused patients (general ward care or delayed intensive care)
d) varying degree of adjustment for confounders, case-mix of the study populations and different ICU settings, and
e) different hospital discharge policies across centres and settings.

Effect of ICU admission on short-term mortality: modifying the SAPS II model
Since we had access to individual risk profiles as expressed by SAPS II, it was appropriate to take individual heterogeneity of risk and probable effects into account. Unfortunately, we did not have information about diagnoses or reason for ICU admission in our study population. Shmueli and Sprung demonstrated how the in-hospital survival benefits of ICU care differ across APACHE II scores and diagnoses [11]. We made the assumptions a) to d) stated on page 8 in the main article text to approximate the published data and cover the relevant ranges of short-term survival benefits reported in Table 1. Adjusting the SAPS II model could do this.

We modified the logit function of the original SAPS II by choosing a *ß1* = 0.09 (range 0.0737 to 0.14 in probabilistic sensitivity analysis). We used the modified SAPS II model to predict the risk of death of NIR patients hypothetically refused admission ("General ward" in Figure 5) [12]. The difference in predicted risk between the modified SAPS II model and the updated SAPS II model calibrated to our study population ("ICU" in Figure 5) express the short-term survival benefit of ICU admission vs. rejection [1]. In Figure 5, the survival benefits are expressed in absolute and relative terms according to SAPS II.

Any gain in QALYs from ICU admission was assumed to be the result of the reduction in the short-term risk of death attributed to the decision to admit to the ICU compared with the hypothetical rejection of ICU admission.

In probabilistic sensitivity analysis, we assumed that the counterfactual ward patient had a risk of death as predicted by range from a) a modified SAPS II model with *ß1* = 0.14 ("General ward max"), to b) the original SAPS II model with *ß1* = 0.0737 published in 1993 (Figure 5).

Table 1 Unadjusted mortality of patients admitted vs. refused ICU and adjusted effect estimates reported in selected studies

Reference	Patient population and country	Reasons for refusal	No of deaths WITH ICU admission	% mortality WITH ICU admission	No of deaths WITHOUT	% mortality WITHOUT or delayed ICU admission	Follow-up	Absolute difference in short-term death risk (%)	Relative risk ratio	Reported effect of adjusted analysis	Remark
Robert 2012	General, 10 ICUs in western France	Delay due to full ICU	276/1136	24.3	58/193	30.1	28 day	5.8	0.81		Those refused admission if too well/too sick were excluded
Louriz 2012	General, single centre, Marocco	Too well, too sick, full ICU, more data needed	37/110	33.3	70/142	49.3	Hospital	16.0	0.68		Left out those with delayed admission
Sprung 2012	General and speciality, 11 centres in 7 European countries	Too well, too sick, no ICU beds available					28 day				ELDICUS, part II, table 4
Age groups	18-44			10.2		12.5		2.3	0.82	OR = 0.74 when age ≤65	
	45-64			21.2		22.3		1.1	0.95		
	65-74			27.9		34.6		6.7	0.81	OR = 0.65 when age >65	
	75-84			35.5		40.4		4.9	0.88		
	85+			41.5		58.5		17.0	0.71		
Ridley 2007 (review, 7 studies)	General, France, UK, Israel, Hong Kong	too sick, too well or no beds available	614/2042	30.1	547/1145	47.7	Hospital	17.6	0.63	OR = 2.09 (of non-admission)	Random effects pooled estimate
Shmueli 2005	General, single centre, Israel			15		43	Hospital	28.0	0.35	Average predicted benefit: ARR 21.0 %	

Abbreviations: OR: odds ratio; ARR: absolute risk reduction

Fig 5 Short-term survival benefit and SAPS II: assumptions

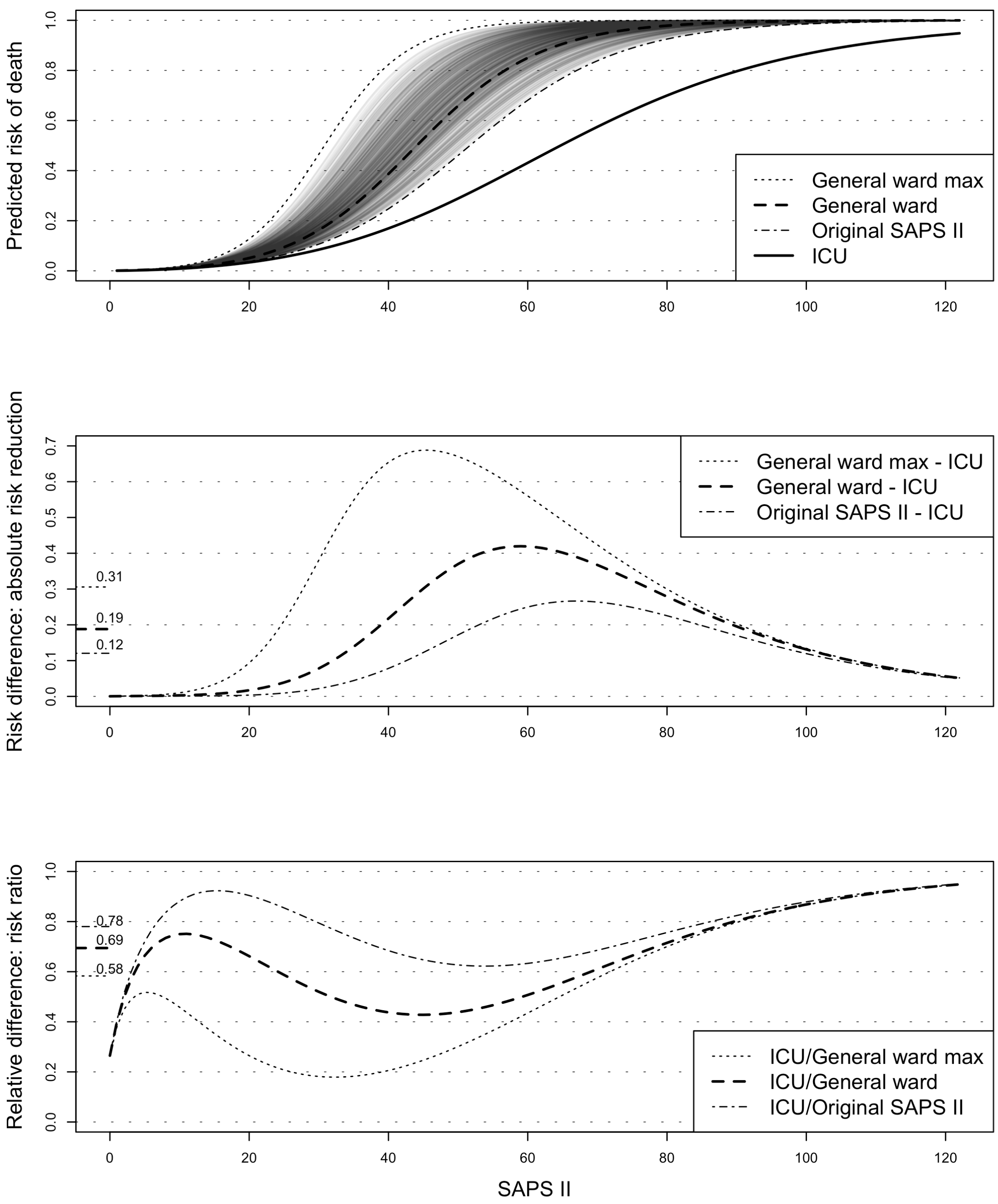

Top: SAPS II vs. mortality as predicted by the calibrated (“ICU”), original, modified base case (“General ward”) and modified maximum (“General ward max”) SAPS II models.

The short-term survival benefit of ICU admission was the mortality difference between the sampled modified model (“shadow” from the range original SAPS II to general ward max) and the calibrated model.

Middle: Absolute difference according to SAPS II (mean=0.19 in base case, between 0.12 and 0.31 in probabilistic sensitivity analyses).

Bottom: Relative risk ratio according to SAPS II (mean=0.69 in base case, between 0.58 and 0.78 in probabilistic sensitivity analyses)

Costs

Table 3 Preliminary data from estimation of costs (NOK) per patient in Norwegian hospitals

Hospital, Norway	Total costs*		Year	Remark	Reference
	Mean per ICU day (NOK)	Mean per general ward day (NOK)			
Innlandet hospital trust	79 032	8 899	2015	Excluding ancilliary services such as laboratory and imaging services	Personal communication, Kjell Nordaune
University Hospital of North Norway (UNN), Tromsø	35 381		2015	Salary = 33 516. Overhead = 1 865. Excluding specific procedures and associated medication and equipment, such as mechanical ventilation, or ECMO, which is costed at 75 100 at the start of a treatment episode.	Personal communication, Stig Bakken
Vestfold Hospital Trust	53 163	5 303	2016	The estimate for a general ward is a mean from various medical wards and orthopedic ward (range NOK 4469-6604)	Personal communication, Torgeir Grøtting
		10 803		Emergency ward observation unit	
		25 929		Medical monitoring unit	
		5 614		Rehabilitation ward (mean from cancer, surgical, neurological and physical rehabilititaion wards (range NOK 5311-6177)	
Østfold Hospital Trust	52 723	5 578	2017	1st quarter. The estimate for a general ward is a mean from various medical and surgical wards (range NOK 4505-6351)	Personal communication, Karine Løllke
		10 955		Observation and acute surgery unit	
		15 999		Postoperative unit	
		32 822		Medical monitoring unit	

* Total costs include fixed costs such as nurse and physician salary and overheads of running the hospital, and variable costs such as medication and disposables in Norwegian kroner (NOK).

The national cost per patient specification (CCP) aims to assign all accounting costs over a time period to patients who received services in that period. CCP is based on the principles of Time Driven Activity Based Costing [13].

The variation in cost estimates between hospitals can partly be explained by local adaptation of the national cost per patient specification. We chose a mean cost of an ICU day of NOK 50 000 (range 30 000 – 70000) based on these data. Our choice of modelling average ward day cost as NOK 8000 (4000 – 12000) was based also based on these data, plus 1) the assumption that treating the critically ill in a ward setting would probably attract resources to the most advanced functions. Hospitals deal with levels of care below high level ICU (multi-organ support) differently, therefore we chose a mean from the higher range of reported data., and 2) the fact that in 2001, the ratio of the cost per ICU day to hospital bed-day was estimated to be six (this is the latest study of the cost of an ICU bed-day in Norway available) [14]. The ratio here would be 50 000/8 000 = 6.25.

Distribution-sensitive weighting function

What it does, and why

We applied a distribution-sensitive weighting function that increases the denominator of the ICER by assigning higher weight to health gains the less expected lifetime QALYs of the recipient. When the incremental costs are fixed, the resulting ICER decreases with fewer lifetime QALYs. Using weighted ICERs therefore implies that we would accept higher costs per unit of health gain for those with more severe conditions. The distribution-sensitive weighting function provides an explicit and systematic way to build into the CEA the trade-off between efficiency and concern for severity of disease in terms of expected lifetime health without intervention [15-17].

How much extra weight to severity of disease? Applying the distribution weights.

The third Norwegian Committee on Priority Setting in the Health Sector suggested that we could accept up to three times higher costs per unit of health gained for high-severity patients (lifetime QALYs < 50) compared to low-severity patients (65+ lifetime QALYs) (1-2-3 rule, a staircase model of cost-effectiveness thresholds) [18]. The distribution-sensitive weighting function was calibrated to fit the 1-2-3 rule. The function multiplies a QALY gain with 1 at 80 lifetime QALYs, with 2 at 65 lifetime QALYs, and with 3 at 50 lifetime QALYs. The function draws a straight line through these points. The area under the curve gives the total weight for a specific QALY gain (Figure 6)[19]. We used undiscounted lifetime QALYs as a basis for defining the distribution weights for a given patient. In principle, we should therefore also have used undiscounted health gains, but this approach is not widely accepted in the literature.

Fig 6 Distribution-sensitive weighting function and lifetime QALYs

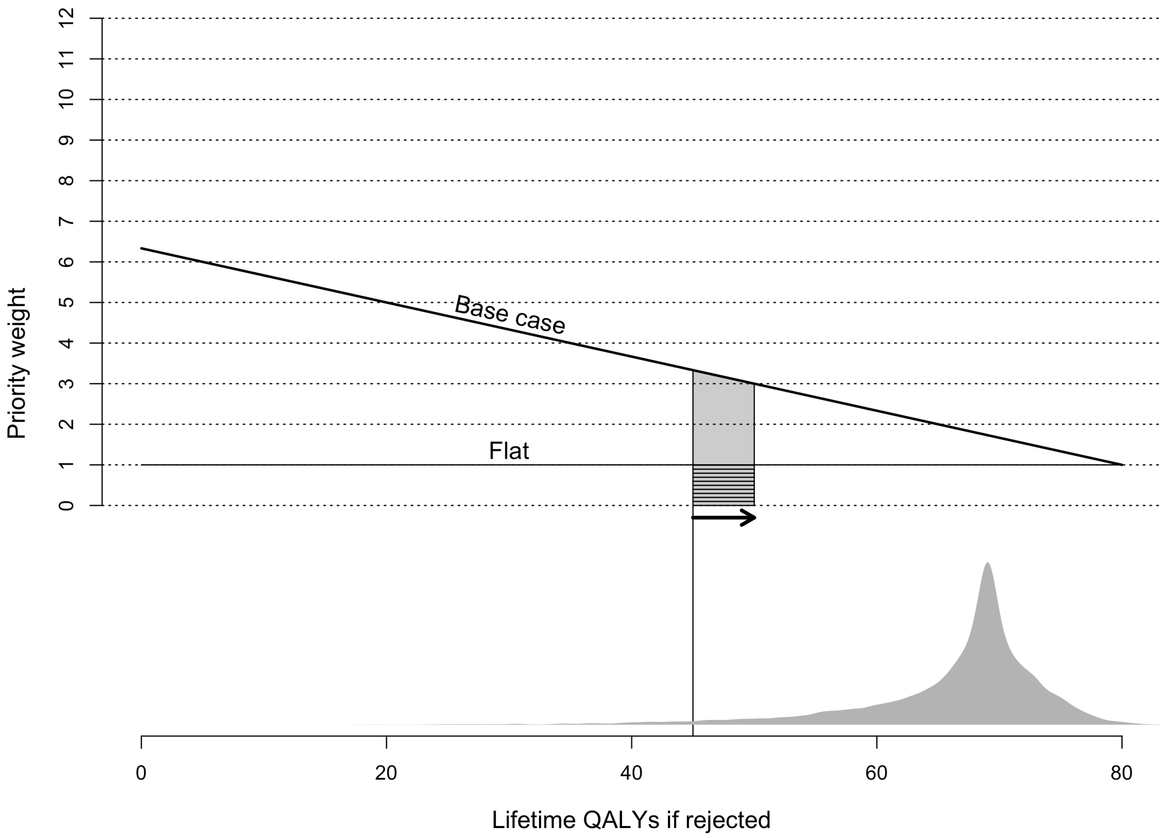

Illustration of the distribution-sensitive weighting function (top) and the distribution of the estimated individual lifetime QALYs in the study population if not admitted to ICU (bottom). When the lifetime QALYs are estimated to be 45 QALYs, a health gain of 5 QALYs from ICU admission (indicated by the thick arrow) will be weighted as follows: Weighted health gain = 5 x the grey area under the weighting curve “Base case” = 15.83.

1. Haaland OA, Lindemark F, Flaatten H, Kvale R, Johansson KA: **A calibration study of SAPS II with Norwegian intensive care registry data**. *Acta Anaesthesiol Scand* 2014, **58**(6):701-708.
2. Lindemark F, Haaland OA, Kvale R, Flaatten H, Johansson KA: **Age, risk, and life expectancy in Norwegian intensive care: a registry-based population modelling study**. *PLoS One* 2015, **10**(5):e0125907.
3. Flaatten H, Kvale R: **Survival and quality of life 12 years after ICU. A comparison with the general Norwegian population**. *Intensive Care Med* 2001, **27**(6):1005-1011.
4. Cuthbertson BH, Roughton S, Jenkinson D, Maclennan G, Vale L: **Quality of life in the five years after intensive care: a cohort study**. *Crit Care* 2010, **14**(1):R6.
5. Sprung CL, Baras M, Iapichino G, Kesecioglu J, Lippert A, Hargreaves C, Pezzi A, Pirracchio R, Edbrooke DL, Pesenti A *et al*: **The Eldicus prospective, observational study of triage decision making in European intensive care units: part I--European Intensive Care Admission Triage Scores**. *Crit Care Med* 2012, **40**(1):125-131.
6. Ridley S, Morris S: **Cost effectiveness of adult intensive care in the UK**. *Anaesthesia* 2007, **62**(6):547-554.
7. Talmor D, Shapiro N, Greenberg D, Stone PW, Neumann PJ: **When is critical care medicine cost-effective? A systematic review of the cost-effectiveness literature**. *Crit Care Med* 2006, **34**(11):2738-2747.
8. Sinuff T, Kahnamoui K, Cook DJ, Luce JM, Levy MM: **Rationing critical care beds: a systematic review**. *Crit Care Med* 2004, **32**(7):1588-1597.
9. Louriz M, Abidi K, Akkaoui M, Madani N, Chater K, Belayachi J, Dendane T, Zeggwagh AA, Abouqal R: **Determinants and outcomes associated with decisions to deny or to delay intensive care unit admission in Morocco**. *Intensive Care Med* 2012, **38**(5):830-837.
10. Robert R, Reignier J, Tournoux-Facon C, Boulain T, Lesieur O, Gissot V, Souday V, Hamrouni M, Chapon C, Gouello JP: **Refusal of intensive care unit admission due to a full unit: impact on mortality**. *Am J Respir Crit Care Med* 2012, **185**(10):1081-1087.
11. Shmueli A, Sprung CL: **Assessing the in-hospital survival benefits of intensive care**. *Int J Technol Assess Health Care* 2005, **21**(1):66-72.
12. Le Gall JR, Lemeshow S, Saulnier F: **A new Simplified Acute Physiology Score (SAPS II) based on a European/North American multicenter study**. *JAMA* 1993, **270**(24):2957-2963.
13. Helsedirektoratet: **Nasjonal spesifikasjon for KPP-modellering 2012**. In. Oslo, Norway; 2012.
14. Flaatten H, Kvale R: **Cost of intensive care in a Norwegian University hospital 1997-1999**. *Crit Care* 2003, **7**(1):72-78.
15. Magnussen J: **På ramme alvor: Alvorlighet og prioritering. Rapport fra arbeidsgruppe nedsatt av Helse- og omsorgdepartementet**. In. Oslo: Helse- og omsorgsdepartementet; 2015.
16. Minstry of Health and Care Services: **Åpent og rettferdig - prioriteringer i helsetjenesten. NOU 2014:12.** In: *Norges offentlige utredninger; NOU 2014:12.* 2014: 218 s.
17. Brock DW: **Priority to the worse off in health-care resource prioritization**. In: *Medicine and Social Justice: Essays on the Distribution of Health Care.* Edited by

Rhodes R, Battin MP, Silvers A. New York: Oxford University Press; 2002: 362-372.

18. Ottersen T, Forde R, Kakad M, Kjellevold A, Melberg HO, Moen A, Ringard A, Norheim OF: **A new proposal for priority setting in Norway: Open and fair**. *Health Policy* 2016.
19. Barra M, Rand-Hendriksen K: **A missing cornerstone in the Norwegian Priority Commission's weighting scheme**. *Nordic Journal of Health Economics Online ISSN* 2015, **1892**:971.
